# Gender Differences in Medication Adverse Effects Experienced by People Living With Chronic Pain

**DOI:** 10.3389/fpain.2022.830153

**Published:** 2022-05-10

**Authors:** Hermine Lore Nguena Nguefack, M. Gabrielle Pagé, Line Guénette, Lucie Blais, Mamadou Diallo, Marimée Godbout-Parent, Adriana Angarita-Fonseca, Anaïs Lacasse

**Affiliations:** ^1^Département des Sciences de la Santé, Université du Québec en Abitibi-Témiscamingue, Rouyn-Noranda, QC, Canada; ^2^Centre de Recherche du Centre Hospitalier de l'Université de Montréal, Montréal, QC, Canada; ^3^Département d'anesthésiologie et de Médecine de la douleur, Faculté de médecine, Université de Montréal, Montréal, QC, Canada; ^4^Faculté de Pharmacie, Université Laval, Québec City, QC, Canada; ^5^Centre de Recherche du Centre hospitalier universitaire (CHU) de Québec – Université Laval, Québec, QC, Canada; ^6^Faculté de Pharmacie, Université de Montréal, Montréal, QC, Canada

**Keywords:** sex, gender, chronic pain, adverse effects, side effects

## Abstract

**Objectives:**

Understanding gender differences in chronic pain (CP) outcome research is essential to optimal treatment delivery. This study explored the associations between gender identity, gender roles, and the number of non-life-threatening pain medication adverse effects reported as severe by people living with CP.

**Methods:**

The analyses were conducted using the COPE Cohort, a dataset generated through a web-based recruitment of adults with CP. Participants were asked how they identified themselves (women, men, unknown, unspecified) and gender roles were measured using the Bem Sex-Role Inventory (subgroups were formed applying the median split method). Pain medication adverse effects were assessed using a standardized checklist (none/mild/moderate/severe). A zero-inflated Poisson model was used to assess gender identity, gender roles and their interaction as potential predictors of the number of pain medication adverse effects.

**Results:**

A total of 1,343 participants reported using pain medications. Adjusting for potential confounders, both gender identity (men vs. women: ß = −0.32, *p* = 0.0024) and gender roles (androgynous vs. undifferentiated: ß = 0.26, *p* = 0.0030) were associated with the number of pain medication adverse effects reported as severe, and they interacted with each other. The stratified analysis by gender roles showed that women reported a greater number of severe adverse effects than men among those classified as masculine and androgynous.

**Discussion:**

Although we are unable to confirm whether the associations can be explained by differences in the experience or in the reporting of effects, gender identity and gender roles should both be explored when studying pain medication adverse effects.

## Introduction

While various non-pharmacologic methods are recommended for the management of chronic pain (CP) (1–3), medications are still used by the majority of patients (62–84%) (4–6). Since non-life-threatening adverse effects related to medication may lead to non-adherence to treatment, suboptimal effectiveness, impaired quality of life and an increased use of healthcare resources (7, 8), a thorough assessment of the adverse effects of pain medication is most warranted. When patients weigh adverse effects against symptom reduction, up to 40% report that adverse effects are more important or as important (9).

The efficacy and adverse effects of drugs are assessed individually during randomized controlled trials (RCT). However, these gold-standard studies are often conducted under strict conditions insomuch that the scope of their conclusions is minimal in the real-world context (10). Indeed, certain patient groups are underrepresented (e.g., patients using more than one drug or with multiple comorbidities) (11, 12). Previous studies revealed that few patients in the community would meet the inclusion criteria of major RCTs in their therapeutic field (0–36%) (13, 14). In addition, for a long time women were underrepresented in RCTs (15). Thus, studying the real-world risks of pain pharmacotherapy is important, especially in a context where CP treatment is characterized by polypharmacy, off-label prescribing and use, and multimorbidity (16–22). Two paramount avenues can thus be explored to help inform and prioritize prevention and development of support tools for patients: (1) providing a real-world picture of the most problematic non-life-threatening pain medication adverse effects among persons living with CP, and (2) identifying individuals most at risk of pain medication adverse effects.

In this light, one may wonder how women, men and gender-diverse people may be differentially impacted by pain medication adverse effects. In contrast to sex, which can be defined as a set of biological attributes associated with physical/physiological features (23), gender refers to socially constructed roles, behaviors, expressions and identities of girls, women, boys, men, and gender diverse people (23). Various gender constructs can help understand this multidimensional concept: 1) gender identity (how individuals see themselves—e.g., man, woman, non-binary, two-spirited), 2) gender roles (behavioral norms applied to males and females that influence everyday actions, expectations and experiences), 3) gender relationships (how individuals interact with and are treated by others based on their gender), and 4) institutionalized gender (distribution of power between men and women in the institutions of society) (24).

In acute and chronic pain populations, studies showed that women are more likely than men to experience pain medication adverse effects (e.g., nausea, vomiting, skin problems, nervous system issues) (25–27). Women are also more likely to stop their pain treatment because of adverse effects (28). However, without proper measurement/consideration of gender constructs, it is questionable whether those associations are explained by biological and/or social factors. To our knowledge, no studies explored how gender identity and gender roles interact to affect adverse effects of pain medication. This study aimed to describe the most frequent non-life-threatening adverse effects of pain medication reported by persons living with CP and explore the associations between gender identity, gender roles, their interaction and the number of adverse effects reported as severe by participants.

## Materials and Methods

### Study Setting and Data Source

This retrospective study was conducted using data from the ChrOnic Pain trEatment (COPE) Cohort (29), a dataset intended to better understand the real-world utilization of pharmacological, physical and psychological treatments among people living with CP. The COPE Cohort was implemented in the province of Quebec (Canada) and includes 1,935 adults living with CP who completed a web-based questionnaire between June and October 2019. Pain duration was self-reported in the questionnaire in years, months days, and the International Classification of Diseases 11th Revision CP definition was applied in terms of eligibility [i.e., persistent or recurrent pain for more than 3 months (30)].

In order to better understand the particularities of our study setting, it is important to give some precision on the endorsement of gender identities and roles in the Canadian society and more precisely, in Quebec which is the only province where French is the majority and the sole official language. Quebec is a society that increasingly values the affirmation of gender identity (31). There has been in the last 4 decades a rapid evolution in terms of women's emancipation, their role in the workforce and high levels of decision-making (32–34). The involvement of men in traditionally feminine roles such as childcare and household management is also increasingly valued (35, 36).

The COPE Cohort self-reported questionnaire included all indicators identified as a minimum dataset by the Canadian Registry Working Group of the Strategy for Patient-Oriented Research (SPOR) Chronic Pain Network (CPN) (37). Item selection was also guided by core outcome domains and measures identified by the Initiative on Methods, Measurement and Pain Assessment in Clinical Trials (IMMPACT) (38, 39), items of the Canadian minimum dataset for chronic low back pain research (40), and variables assessed in the Quebec Pain Registry (41). Self-reported COPE data was also intended to be linked to longitudinal administrative data (medical and prescription claims). The complete methodology of the COPE Cohort implementation is described elsewhere (29). The study protocol was approved by the Université du Québec en Abitibi-Témiscamingue's research ethics committee and informed consent was obtained from all participants. This study was conducted using the self-reported data and among the sample of participants who reported using prescribed and/or over-the-counter (OTC) medication to treat their pain (*n* = 1,343).

### Study Variables

The number of pain medication adverse effects reported as severe by participants was considered the primary outcome, with gender identity and gender roles as the main independent variables of interest.

#### Pain Medication Adverse Effects

Among the COPE Cohort participants, adverse effects related to pain medication were self-reported using a standardized list of 19 adverse effects common with these medications that was previously used in the Canadian Neuropathic Pain Database (42) and the Quebec Pain Registry (41): lightheadedness/dizziness, drowsiness, confusion, nausea, vomiting, impaired memory, dry mouth, itching, abdominal discomfort, constipation, slowing of the urine stream, fatigue, insomnia, swelling, weight gain, visual blurring, decreased sex drive, hallucinations, nightmares. Participants were asked “*Are you experiencing any of the following side effects from your current treatment for pain?”* and rated presence and intensity (none/mild/moderate/severe) of each effect. Hence, the number of pain medication adverse effects reported as severe could be computed for each participant.

#### Gender Identity and Gender Roles

In the questionnaire, participants self-identified as women, men, unknown or unspecified using the gender item of the National Institutes of Health (NIH) Task Force on Research Standards for Chronic Low Back Pain Minimum Dataset (43). According to recent literature, women vs. men self-identification reflects sex assigned at birth in more than 97% of cases (0.1–2.3% of large survey respondents in Canada, the US, and Europe report that the sex assigned at birth differs from their current gender identity—e.g., transgender, non-binary, two-spirit) (44, 45). Gender roles were measured using the Bem Sex-Role Inventory (BSRI) (46) [18-item French version (47)]. The BSRI assesses stereotypically feminine and masculine self-described personality traits (48). Although it was criticized by various authors (e.g., stereotypically feminine and masculine traits that have evolved over the last few decades), it is the most used instrument in gender research literature (48), Since its first publication in 1974 (60 items) (46), several versions and scoring methods were used over the years that appear to be valid across countries and cultures. The 18-item French version published by Fontayne et al. (47) was chosen because it is brief and deemed appropriate for people with various literacy levels [as items were found to be understood by teenagers (47)]. Each item is scored on a 7-point Likert scale ranging from “1 = Never true” to “7 = Always true” (46) and they are averaged to obtain a feminine score (10 items) and a masculine score (8 items) (47). These two scores can then be used to create four gender role subgroups using the median-split approach and the sample median (49, 50): 1) participants scoring higher than or equal to the feminine scale median and below the masculine scale median are categorized as having a “feminine” profile, 2) those scoring below the feminine scale median and higher than or equal to the masculine scale median are categorized as having a “masculine” profile; 3) those scoring higher than or equal to the median on both scales are classified as “androgynous,” and 4) those scoring below the median on both scales are classified as “undifferentiated”. Based on the theoretical model of this version of the BSRI (47), participants categorized as “feminine” have a greater tendency to describe themselves as tender and sensitive to others; participants categorized as “masculine” rather describe themselves as athletic, having leadership, and being self-confident; participants are categorized as “androgynous” when scoring high on all these traits and as “undifferentiated” when scoring low on all these traits. The classification must therefore be interpreted based on that logic. In our sample of CP adults (members of the COPE Cohort), the internal consistency and factor structure of this short version of the BSRI were shown adequate, i.e., Cronbach's alphas of 0.90 [95% confidence intervals (95% CI) = 0.89–0.91] and 0.82 (95% CI = 0.81–0.84) were obtained for the feminine and masculine scales, respectively; confirmatory factor analysis reproduced the five first-order factors (tenderness, sensitivity to others, athletic, leadership, self-confidence) and two second-order factors (feminine, masculine) of the theoretical model published by Fontayne et al. (47) with acceptable goodness of fit indices ($\chi_{\left(125\right)}^{2}$ = 1,202.62 *p* < 0.0001, GFI = 0.9008, CFI = 0.9147, RMSEA = 0.0823).

#### Chronic Pain-Related Variables

The location of pain in the body was operationalized as dichotomous non-mutually exclusive variables. For this study, the five most frequently reported locations in the sample (i.e., back, neck, shoulders, legs, hips; yes/no) were described, in addition to the presence of generalized pain (yes/no) and multisite pain (i.e., two or more locations). The following aspects were also considered: 1) the circumstances surrounding the onset of pain, 2) pain duration, 3) frequency, 4) intensity (0–10 numerical rating scale measuring the average pain intensity over the past seven days), 5) pain catastrophizing [single item of the NIH Minimum dataset (43) and STarT Back Screening Tool (51)], 6) the neuropathic component of pain [DN4 Interview part; a score ≥3/7 indicates a likely presence of neuropathic pain (52)], and 7) pain interference [Brief Pain Inventory (BPI) Interference Scale (53) which ranges from 0 to 10].

#### Pain Treatment

In the COPE questionnaire, current use of prescribed medications, OTC medications, and physical/psychological treatments used for pain management were defined as dichotomous variables (yes/no). The percentage of relief provided by pain treatment was self-reported on a numeric scale ranging from 0% (no relief) to 100% (complete relief). Access to a trusted healthcare professional for pain management and the total number of medications currently used (including prescribed, OTC, pain-related and other disease-related medications) were also self-reported by COPE Cohort participants. Longitudinal administrative data [private and public insurance prescription claims obtained through the reMed registry (54)] were linked to questionnaire data for a portion of participants. The detailed pharmacotherapy profile (i.e., specific drugs used in the year before and the year after the completion of the questionnaire) was thus available for 152 participants. All reMed data access requirements and ethical authorizations were obtained.

Other covariates measured in the COPE Cohort (29) and included in the present study were sociodemographic characteristics and health profile (physical functioning, general health, psychological distress, substance abuse, smoking status, use of cannabis).

### Statistical Analysis

A sex- and gender-based analysis was conducted (55–57), including stratified statistics, statistical significance of gender identity (in a way a proxy for sex at birth in the present study), gender roles and their interaction terms in multivariable models, and reporting of negative findings (statistically non-significant results). First, descriptive statistics (means, standard deviations, counts, percentages) were used to summarize the main characteristics of the whole study population in addition to their adverse effects profile (most commonly reported pain medication adverse effects; overall and those reported as severe by participants). The distribution of gender role subgroup membership was also described across gender identity categories. Bivariate analyses (Chi-square, Wilcoxon rank-sum and Kruskal-Wallis tests) were used to assess gender identity and gender roles differences regarding: (1) the number of pain medication adverse effects reported as severe by participants, (2) the proportion of participants without adverse effects, (3) the prevalence of the four most frequently reported adverse effects regardless of their severity (fatigue, dry mouth, drowsiness, decreased sex drive), and (4) the prevalence of the four most common adverse effects reported as severe (fatigue, decreased sex drive, dry mouth, insomnia).

A multivariable two-part regression model (58) was used to assess the association between gender identity and gender roles (independent variables), and the number of pain medication adverse effects reported as severe by participants (dependent variable).

A complete description of the two-part modeling is presented in Supplementary Digital Content 1. Results of the first part of the model (logistic regression) were computed as adjusted odds ratios (OR) along with their respective 95% CI and *p*-values; results from the second part of the model (Poisson regression) were computed as adjusted beta coefficients (ß) along with their respective 95% CI and *p*-values. All variables measured in the COPE Cohort that could potentially be associated with gender identity, gender roles or adverse effects of medications (potential confounders) were identified *a priori* and included in the regression analysis. The choice of variables was based on existing literature and clinical considerations. Because of our substantial sample size, this approach was chosen over other criticized selection techniques such as relying on bivariate regression analyses *p*-values (59) or on computer algorithms (60). Variance inflation factors (VIFs) below 5 (61) were used to detect variables showing a multicollinearity problem. Interaction terms (*gender identity* ^*^ *gender role* dummy variables) were tested. In case of statistical significance, it was planned to better map and evaluate the direction of effect modification by stratifying the gender identity multivariable regression coefficient across gender role subgroups.

Although the total number of medications used by participants was self-reported (regardless of their indication), the detailed types and posology of medications used by participants were not collected in the COPE Cohort self-administered web-based questionnaire, thus making it impossible to test high-risk medications (e.g., opioids, benzodiazepines) as potential confounders or effect modifiers of gender identity- and gender- associations. However, having access to private and public insurance prescription claims for a small portion of the cohort, it was possible to conduct a sensitivity analysis regarding the robustness of our conclusions (multivariable model including only gender identity, gender role dummy variables, opioid and benzodiazepine use). Dispensed prescriptions were classified according to the Anatomical Therapeutic Chemical (ATC) classification system for opioids (N02A) and benzodiazepines (N05BA, N05CD, N03AE, and benzo-related drugs N05CF). A user was then defined as a participant who was dispensed such drugs in the 90 days before the completion of the questionnaire (to account for the refill gap period). A sensitivity analysis was also carried out to assess the impact of missing value imputation on conclusions. A multiple imputation approach was used as suggested in the literature (60). All analyses were performed using SAS^®^ version 9.4 (SAS Institute, Cary, NC, USA).

## Results

A total of 1,433 participants completed the section of the questionnaire about pain relief strategies. Of those, 1,343 (93.72%) reported using pain medications (28.58% used prescribed medications only, 15.00% OTC medications only, 56.42% used both), which formed the convenience sample for our study.

Table 1 shows the characteristics of the study participants. Age ranged between 18 and 88 years (mean: 50.06 ± 13.14), and 84.64% were women. Four participants identified as non-binary (it was thus impossible to form a statistically sound subgroup for all of our analyses). Regarding gender role subgroups, 26.99% were classified as feminine (described themselves as tender and sensitive to others), 19.53% as masculine (described themselves as athletic, having leadership, and being self-confident), 30.85% as androgynous (scored high on all these traits), and 26.99% as undifferentiated (scored low on all these traits). Over half of the participants (52.16%) had been suffering from pain for at least 10 years, and the most common pain location was the back (63.22% of participants). Only 35.05% reported being employed (full- or part-time) and 79.29% had post-secondary education.

**Table 1:** Sample characteristics.

**Characteristics (*n* = 1,343)**	No. (%) of participants^*^
**Age (years)**—mean ± SD	50.06 ±13.14
**Gender identity**	
Women	1,119 (84.64)
Men	199 (15.05)
Unknown/undetermined	4 (0.30)
**Gender role subgroups**	
Feminine	270 (26.99)
Masculine	233 (19.53)
Androgynous	368 (30.85)
Undifferentiated	322 (26.99)
**Pain frequency**	
Continually	1,174 (87.81)
Occasionally	163 (12.19)
**Pain duration (years)**	
<1	44 (3.06)
1–4	298 (22.24)
5–9	302 (22.54)
≥10	699 (52.16)
**Pain intensity on the average in the past 7 days (0–10)**—mean ± SD	5.47 ± 1.93
**Pain intensity at its worst in the past 7 days (0–10)**—mean ± SD	7.32 ± 1.71
**Most common pain locations**	
Back	849 (63.22)
Neck	614 (45.72)
Shoulders	593 (44.15)
Legs	530 (39.46)
Hips	516 (38.42)
**Country of birth**	
Canada	1,253 (96.09)
Other	51 (3.91)
**Employment**	
Worker	457 (35.05)
Unemployed	847 (64.95)
**Education level**	
Post-secondary education	1,030 (79.29)
No post-secondary education	269 (20.71)

* *Unless stated otherwise*.

*Proportion of missing data across presented variable ranges between 0 and 11.17%*.

*SD, Standard deviation*.

**Figure 1:**
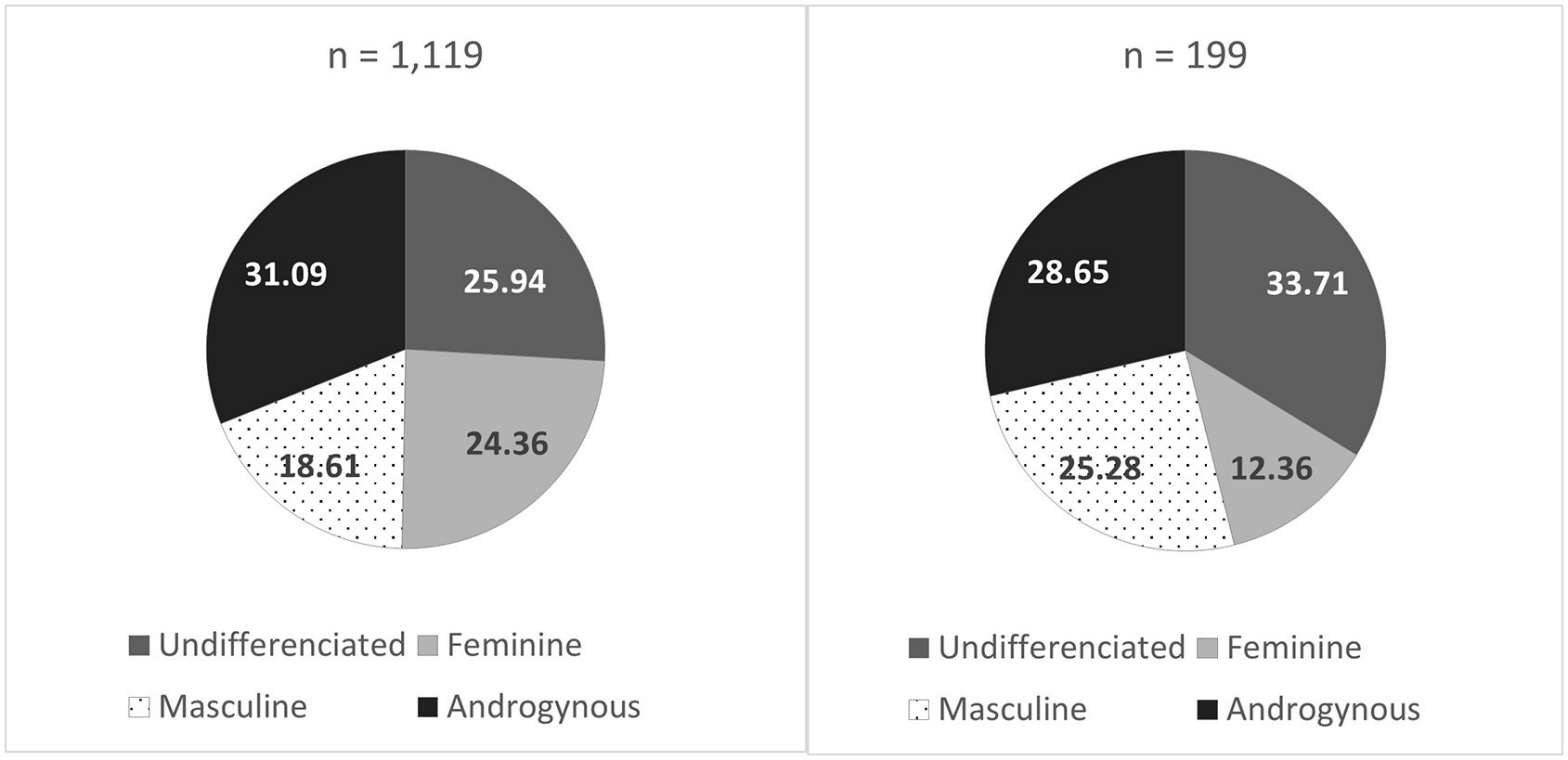
Distribution (%) of gender role subgroups in women (left) and men (right). Feminine: described themselves as tender and sensitive to others. Masculine: described themselves as athletic, having leadership, and being self-confident. Androgynous: scored high on all these traits. Undifferentiated: scored low on all these traits.

The distribution of the four gender role classifications among women and men is presented in Figure 1. Among women, 24.36% were classified as having feminine gender roles, 18.61% as masculine, 31.09% as androgynous and 25.94% as undifferentiated. Among men, these proportions were, respectively, 12.36, 25.28, 28.65, and 33.71%.

### Most Frequent Adverse Effects of Pain Medication

**Figure 2:**
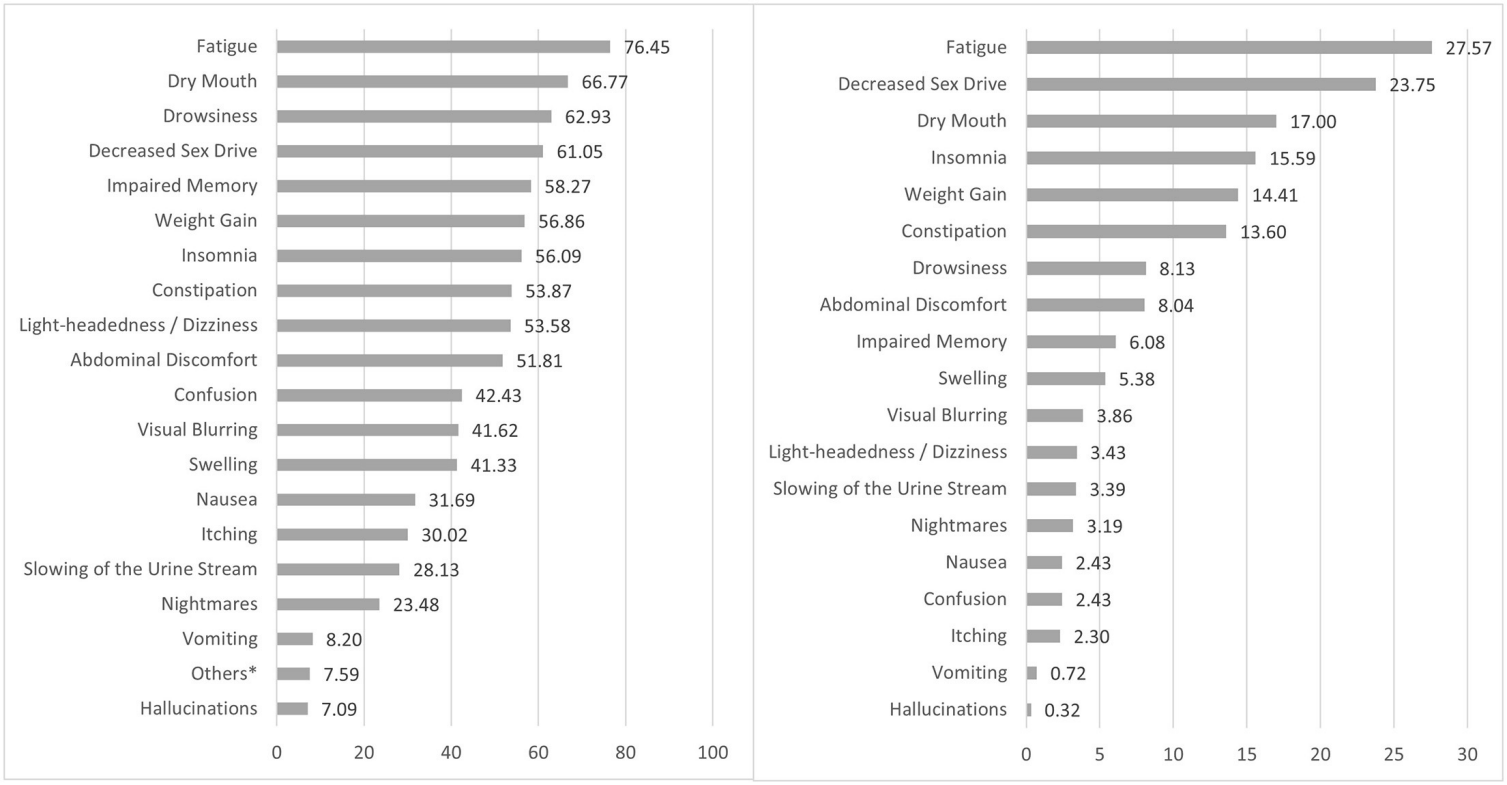
Most frequently reported adverse effects (left) and adverse effects reported as severe (right). * Other adverse effects reported in the open-ended question at the end of the standardized checklist included stomach burn (12.75%), night sweating and hot flashes (10.78%), pain (5.88%), mood swing (4.90%), and lack of appetite (3.92%).

Regardless of their severity, the four most frequently reported pain medication adverse effects among the study population were fatigue (76.45%), dry mouth (66.77%), drowsiness (62.93%) and decreased sex drive (61.05%) (Figure 2). The most common adverse effects reported as severe by participants were fatigue (27.57%), decreased sex drive (23.75%), dry mouth (17.00%) and insomnia (15.59%).

### Gender-Stratified Adverse Effects Profile

Only 10.1% of participants reported no pain medication adverse effects. As shown Table 2, bivariate statistical comparisons (that do not control for confounding) mainly showed gender role subgroups differences in terms of the number of reported adverse effects, presence of adverse effects, and prevalence of specific adverse effects (overall and those reported as severe by participants).

**Table 2:** Number of adverse effects reported as severe and most frequently reported adverse effects according to gender identity and gender role subgroups.

	**Total**	**Gender identity subgroups**			**Gender role subgroups**				
	***n*** **= 1,343**	**Men** ***n*** **= 199**	**Women *n* = 1,119**	***p*** -value^*^	**Feminine (F) Describe themselves as tender and sensitive to others *n* = 270**	**Masculine** **(M)** **Describe themselves as athletic, having leadership, and being self-confident** ***n*** **= 233**	**Androgynous (A) Scored high on all these traits *n* = 368**	**Undifferentiated (U)** ***n*** **= 322**	***p*** -value^**^
**Number of adverse effects reported as severe**—mean ± SD	1.56 ± 2.10	1.42 ± 1.96	1.57 ± 2.12	0.5850	2.07 ± 2.42	1.09 ± 1.73	1.46 ± 2.04	1.45 ± 2.05	**<0.0001** *Post-hoc* differences: F-M, F-A, F-U
**Proportion of participants without adverse effects**—*n* (%)	134 (10.10)	13 (6.53)	120 (10.88)	0.0624	21 (7.87)	29 (12.45)	49 (13.39)	24 (7.55)	**0.0270** *Post-hoc* differences: none according to conservative tests
**Most frequently reported adverse effects—*****n*** **(%)**									
Fatigue	990 (76.45)	159 (81.12)	810 (75.42)	0.0842	216 (82.76)	170 (75.56)	257 (71.79)	239 (76.85)	**0.0170** *Post-hoc* differences: F-A
Dry mouth	868 (66.77)	135 (69.23)	715 (66.20)	0.4092	197 (74.62)	147 (64.19)	221 (61.73)	199 (64.40)	**0.0064** *Post-hoc* differences: F-A, F-U
Drowsiness	813 (62.93)	130 (66.67)	667 (62.22)	0.2371	178 (68.20)	142 (62.01)	211 (58.94)	196 (64.05)	0.1217
Decreased sex drive	779 (61.05)	132 (68.75)	629 (59.34)	**0.0140**	179 (68.58)	121 (54.02)	200 (56.82)	194 (63.19)	**0.0029** *Post-hoc* differences: F-M, F-A
**Most frequent adverse effects reported as severe—*****n*** **(%)**									
Fatigue	357 (27.57)	43 (21.94)	306 (28.49)	0.0588	95 (36.40)	46 (20.44)	101 (28.21)	74 (23.79)	**0.0004** *Post-hoc* differences: F-M, F-U
Decreased sex drive	303 (23.75)	51 (26.56)	244 (23.02)	0.2871	76 (29.12)	43 (19.20)	67 (19.03)	87 (28.34)	**0.0022** *Post-hoc* differences: F-A, A-U
Dry mouth	221 (17.00)	29 (14.87)	187 (17.31)	0.4026	62 (23.48)	22 (9.61)	54 (15.08)	48 (15.53)	**0.0004** *Post-hoc* differences: F-M, F-A
Insomnia	197 (15.59)	33 (17.28)	158 (15.06)	0.4353	43 (16.80)	19 (8.56)	63 (18.00)	39 (12.96)	**0.0096** *Post-hoc* differences: F-M, A-M

* *Chi-square tests or Wilcoxon rank-sum tests*.

** *Chi-square tests or Kruskal-Wallis tests with Tukey style multiple comparisons of proportions or Dwass-Steel-Critchlow-Fligner tests for post-hoc pairwise analyses*.

*Proportion of missing data across presented variable ranges between 1.19 and 5.88%*.

*P-values < 0.05 are reported in bold*.

*SD, Standard deviation*.

### Gender Identity and Gender Roles as Predictors of the Number of Adverse Effects Reported as Severe by Participants

Main results of the multivariable model used to assess the association between gender identity, gender roles and the number of adverse effects reported as severe by participants are presented in Table 3. Gender identity and gender roles were both associated with the number of adverse effects reported as severe by participants: (1) Women reported a greater number of severe adverse effects (men vs. women ß: −0.32, 95% CI: −0.52, −0.11), (2) Participants classified as androgynous experienced a greater number of severe adverse effects (androgynous vs. undifferentiated gender roles ß: 0.26, 95% CI: 0.09–0.44). Complete results of the two-part model (including coefficients for all covariables) are presented in Supplementary Digital Content 2.

**Table 3:** Multivariable model exploring associations between gender identity, gender and number of severe adverse effects.

	Adjusted ^*^	**95% CI**	***p*** **-value**
**Coefficients Estimates for the number of severe adverse effects among participants with non-structural zero severe adverse effect**			
**Model without interaction terms**			
**Gender identity** (men vs. women)	**−0.32**	**−0.52 to −0.11**	**0.0024**
**Gender (vs. undifferentiated)**			
Feminine (describe themselves as tender and sensitive to others)	0.06	−0.11 to 0.23	0.4821
Masculine (describe themselves as athletic, having leadership, and being self-confident)	0.03	−0.19 to 0.25	0.7938
Androgynous (scored high on all these traits)	**0.26**	**0.09 to 0.44**	**0.0030**
**Model with interaction terms**			
**Gender identity (men vs. women)**	**-0.58**	**−1.03 to −0.13**	**0.0121**
**Gender (vs. undifferentiated)**			
Feminine	-0.03	−0.21 to 0.16	0.7788
Masculine	0.03	−0.20 to 0.26	0.8000
Androgynous	**0.27**	**0.08 to 0.45**	**0.0042**
**Interaction terms**			
Gender identity ^*^ Feminine	**0.70**	**0.16 to 1.23**	**0.0106**
Gender identity ^*^ Masculine	0.09	−0.61 to 0.78	0.8052
Gender identity ^*^ Androgynous	0.03	−0.52 to 0.59	0.9035

*P-values < 0.05 are reported in bold*.

* *Adjusted for circumstances surrounding onset of pain, pain location, frequency, duration, tendency to pain catastrophizing, evidence of neuropathic pain, Brief Pain Inventory (BPI) score, pharmacological pain treatment use, non-pharmacological treatment use, access to a trusted healthcare professional for pain management, percentage of relief provided by pain treatment, country of birth, employment, disability, education level, living in a remote region, age, physical functioning score, general health score, number of drugs used, Patient Health Questionnaire-4 score, alcohol or drugs perceived problem, cannabis use, and smoking; 976 participants with no missing data were included in the final model*.

When interaction terms between gender identity and gender role dummy variables (feminine, masculine, or androgynous vs. undifferentiated) were tested in the multivariable model (Table 3), statistical significance was reached. Gender identity multivariable regression coefficients were thus disaggregated across gender role subgroups to better map and evaluate the direction of effect modification (Table 4). Gender identity was only associated with the number of adverse effects reported as severe among participants classified as masculine (ß men vs. women: −1.13; 95% CI: −1.78, −0.48) or androgynous (ß men vs. women: −0.81; 95% CI: −1.16, −0.47), in other words, participants who scored high on athletic, leadership, and self-confidence traits.

**Table 4:** Gender-stratified multivariable results.

		Adjusted ^*^	**95% CI**	***p*** **-value**
**Association between gender identity and number of severe adverse effects among gender role subgroups**				
Undifferentiated	Men vs. women	−0.20	−0.68 to 0.27	0.4059
Feminine	Men vs. women	0.28	−0.20 to 0.76	0.2582
Masculine	Men vs. women	**−1.13**	**−1.78 to −0.48**	**0.0007**
Androgynous	Men vs. women	**−0.81**	**−1.16 to −0.47**	**<0.0001**

*P-values < 0.05 are reported in bold*.

* *Adjusted for the same variables listed in Table 3 footnotes*.

As for sensitivity analyses (among participants for which private and public insurance prescription claims were available), only the association between gender roles and the number of adverse effects reported as severe by participants remained significant in the smaller model (*n* = 152) adjusting for opioid and benzodiazepine use (androgynous vs. undifferentiated gender roles ß: 0.73, 95% CI: 0.27–1.19; feminine vs. undifferentiated gender roles ß: 0.68, 95% CI: 0.25–1.11). COPE Cohort participants and the subsample in which sensitivity analyses were conducted were comparable in terms of gender identity (women: 84.64 vs. 83.03%) and gender roles (feminine: 27.00 vs. 28.95%; masculine: 19.53 vs. 17.76%; androgynous: 30.85 vs. 30.92%). Multiple imputation of missing values did not change our main conclusions (the model is presented in Supplementary Digital Content 3).

## Discussion

The present study describes real-world non-life-threatening pain medication adverse effects experienced by individual living with CP and is the first to our knowledge to explore how gender identity and gender roles interact to affect the severity of such effects. When adjustments were made to account for sociodemographic and clinical factors and sufficient statistical power was achieved, both gender identity and gender roles were associated with the number of adverse effects reported as severe. Our results, however, suggest a statistical interaction between those factors, meaning that women had a greater number of adverse effects reported as severe, but only when they presented specific gender profiles.

Based on our results, gender identity and gender roles should not be studied separately and are not interchangeable. In support of the growing recognition of the relevance of sex- and gender-based analysis when studying the experience of pain (62–64), our results emphasize the importance of including both gender identity and gender roles in all CP randomized controlled trials and observational studies about analgesic drug's risks and benefits. Effect modification should also be tested in all studies as we showed that social factors are important. There is still a long way to go when one considers that, all medical fields combined, only 6% of Canadian randomized controlled trials conduct women vs. men subgroup analyses, 4% report sex-disaggregated data, and none operationalize sex and gender variables, nor carry a comprehensive sex- and gender-based analysis (65). Although we are unable to confirm whether the associations are explained by differences in the experience or in the reporting of pain medication adverse effects, gender identity and gender roles should also be considered in knowledge transfer initiatives and clinical practice when trying to prevent, identify and manage pain medication-related adverse effects.

### Most Frequent Adverse Effects of Pain Medication

The most commonly reported adverse effects, regardless of severity, gender identity or gender roles, were fatigue, dry mouth, drowsiness, and decreased sex drive (with prevalence estimates above 60%). Impaired memory, weight gain, insomnia, constipation, lightheadedness/dizziness, and abdominal discomfort also affected more than 50% of participants. Although those effects are non-life-threatening, they remain of great interest as they can have a serious impact on a person's quality of life (7, 8). The content of patient support tools as well as the support offered by healthcare professionals could be focused on these types of adverse effects. Knowing that non-life-threatening adverse effects affect the great majority of patients (90% in our study), healthcare professionals (e.g., physicians, pharmacists, nurse practitioners) should address the subject during consultations to ensure patient informed decisions regarding risks and benefits of using pain medications. It should be noted that since we analyzed a cohort of prevalent analgesic users, our study may even be underestimating the frequency of those adverse events [depletion of susceptibles bias (66)].

### Plausibility of Biological Differences

This study shows that gender identity is associated with the number of pain medication adverse effects reported as severe when adjusting for gender roles, CP characteristics and interference, information about pain treatments, as well as sociodemographic and health profiles. Specifically, women reported a higher number of adverse effects reported as severe than men. This result is consistent with those of other studies that showed that women appear to be more vulnerable than men to certain adverse effects of analgesic drugs (25–28, 67). These results thus raise the following question: Why, biologically speaking, could there be differences in the experience of adverse effects reported as severe between men and women? Related literature speaks of men vs. women differences in synaptic transmission, pharmacokinetics, pharmacodynamics and response to treatment (68, 69). Additionally, it has been shown that the use of both prescribed and OTC analgesics is significantly higher among women than men (70, 71). In general, being a woman has been shown to be a risk factor for clinically relevant adverse effects, with a greater risk of developing an adverse effect compared to male patients (72). For example, in the context of antidepressants, it has been shown that women tend to report more adverse effects, such as dizziness, nausea, vision problems, constipation, and somnolence. Men tend to report greater sexual dysfunction and urinary problems (73, 74). A review also reported that the severity of adverse effects can be more pronounced in women (75).

### Plausibility of Social Differences

This study has revealed an association between gender roles and the number of pain medication adverse effects reported as severe, i.e., participants with androgynous characteristics (those who scored high on all BSRI traits: tenderness, sensitivity to others, athletic, having leadership, being self-confident), reported a greater number of adverse effects reported as severe than those with undifferentiated characteristics. Different potential explanations can be put forward, including differences in verbalization of side effects, coping strategies, perceived severity, and/or importance given to side effects. As traditional gender roles can influence the verbalization of pain (75) they perhaps influence the verbalization of adverse effects. In addition, gender roles are known to be related to coping strategies (76). Personality traits can also be associated with better social support, a factor known to have a positive influence on adjustment to CP (77). An integrative review on gender roles in pain perception and expression showed that femininity seems to be associated with lower pain tolerance thresholds, as well as a greater propensity to report painful sensations (78). Moreover, psychological and social elements of gender have been reported as associated with altered pain experiences and analgesic use profiles. Hence, pain perception may influence analgesic requirements (79), which can in turn affect adverse effects reporting. Our model adjusted for pain characteristics and general information about pain treatments, but further studies are needed to elucidate the causal diagram behind this association. All things considered, our study nevertheless underlines the importance of defining, measuring and including both gender identity and gender in all CP randomized controlled trials and observational studies about drugs risks and benefits. Effect modification should always be explored.

In fact, gender identity and gender interacted to affect the number of adverse effects reported as severe. When stratifying results, gender identity was associated with the number of adverse effects reported as severe among participants with masculine and androgynous characteristics (women reported significantly higher numbers of adverse effects reported as severe than men), but not among participants with feminine and undifferentiated characteristics. This effect modification suggest that although fundamental biological differences may exist between women and men, the experience and/or reporting of adverse effects is shaped by social factors such as personality traits. This underlines the importance of addressing the management of adverse effects using the biopsychosocial model (80), already pronounced in research about CP, but still underused in clinical practice (3).

### Strengths and Limitations

Our study has several strengths such as the diversity and exhaustiveness of the variables considered, the use of many recognized validated scales and its sample size shown to be representative of large random samples of CP adults in terms of pain characteristics, age, employment status, and level of education (29). In the COPE Cohort, women and users of pain medications were however overrepresented. The web-based recruitment methods and questionnaire administration could explain the oversampling of women as they are known to use Facebook (81) and work in online environments more than men (82). Women also use more drugs (83). That said, the sample still allowed us to study both men and women in a diverse spectrum of gender role profiles (Figure 1). In terms of limitations, the COPE Cohort questionnaire did not cover sex assigned at birth. Even if self-identified gender is a good proxy for sex assigned at birth in the general population (44, 45), our interpretations with regards to the influence of sex on adverse effects must be formulated with caution. Excluding participants who self-identified as non-binary (*n* = 4) is ethically problematic, but was justified on grounds of statistical validity. Researchers will have to go beyond the methodology applied in the present study such as exploring the experiences of this subgroup through qualitative approaches or apply more targeted recruitment approaches in large quantitative studies. Too few representatives of racialized subgroups also limited the scope of the sex-, gender- and diversity-based analysis (SGBA+). Further studies should thus be conducted to expand our findings and explore intersectionality. Even if participants were asked about adverse effects related to pain medication, our measure is imperfect as it can sometimes be difficult for patients to know for certain if an adverse effect is caused by pain medications, other medications used to treat comorbidities or the disease itself. At least the patterns of results suggest differences in side effects that are classically medication-related (e.g., dry mouth). We should underline that in the analysis of the BSRI scores, the choice of the median-split method (as opposed to continuous BSRI scores or feminine-masculine difference score) is not without drawbacks (50, 84). In addition, the BSRI only allow an analysis grounded in stereotypical gender roles of the 1980s which might not relate the same way across ages. The BSRI has indeed posed problems of interpretation to researchers in the field of pain (63). Also, we cannot exclude the possibility that observed differences between gender roles subgroups classified according to this inventory could be explained by participants' endorsement of items of the questionnaire (i.e., participants classified as undifferentiated may be more conservative in their self-reports). As for the detailed profile of participants' pharmacotherapy, our analysis is limited (i.e., open to a type II error, do not account for specific types of drugs or dosing) and further studies are needed. Also, we cannot exclude the possibility of a type II error considering that 199 men we included in our sample. Finally, the cross-sectional nature of the study does not enable to establish causal relationships. That said, gender identity and gender roles are probably determining factors as opposed to consequences of adverse effects.

## Conclusion

Despite the limitations of our study, we were able to show that both gender identity and gender roles are associated with the number of pain medication severe adverse effects and interact with each other. Our results emphasize the importance of including both gender identity and gender in all CP randomized controlled trials and observational studies about drugs' risks and benefits. Using a biopsychosocial approach, those factors should be considered when trying to prevent, identify and manage pain medication-related adverse effects.

## Data Availability Statement

The datasets presented in this article are not readily available because participants did not initially provide consent to open data. The data that support the findings of this study are available from the corresponding author (AL) upon reasonable request and conditionally to a proper ethical approval for a secondary data analysis. Requests to access the datasets should be directed to AL, anais.lacasse@uqat.ca.

## Ethics Statement

The study involving human participants was reviewed and approved by Université du Québec en Abitibi-Témiscamingue (UQAT). The patients/participants provided their written informed consent to participate in this study.

## Quebec Consortium on Adverse Effects of Pain Medications

The members of the Quebec Consortium on Adverse effects of pain medications are (in alphabetical order): Aline Boulanger, Anaïs Lacasse, Anne Hudon, Catherine E. Ferland, Céline Gélinas, David Lussier, David Williamson, Émilie Paul-Savoie, Éric Troncy, Gérard Huni, Gilles Lavigne, Graciela Pineyro, Hélène Beaudry, Jennifer Cogan, Kadija Perreault, Laurent Dupuis, Line Guénette, Lise Dassieu, Louis Gendron, M. Gabrielle Pagé, Manon Choinière, Mélanie Bérubé, Nabiha Benyamina Douma, Nancy Julien, Philippe Sarret, Pierre Rainville, Simon Décary, Sylvie Lafrenaye, Sylvie Lemay, and Yoram Shir.

## Author Contributions

AL, MGP, LG, and LB consolidated funding and put in place the web-based COPE Cohort. LB was more specifically involved in the linkage of self-reported data with the reMed prescription claims registry. AL and HN conceptualized this specific subproject and drafted the manuscript. Data analyses were conducted by HN. MD, MG-P, and AA-F brought a significant contribution to the literature review and interpretation of data. All authors revised it critically, gave final approval of the final version, and agreed to act as guarantors of the work. All authors contributed to the article and approved the submitted version.

## Funding

The implementation of the COPE Cohort was supported by the Quebec Network on Drug Research and the harnessing of its data co-funded by the Quebec Pain Research Network, two thematic networks of the Fonds de recherche du Québec – Santé (FRQS).

## Conflict of Interest

AL holds a Junior 2 research scholarship from the FRQS in partnership with the Quebec SUPPORT Unit (Support for People and Patient-Oriented Research and Trials). MGP holds a Junior 1 research scholarship from the FRQS. MG-P and AA-F, respectively, hold Canadian Institutes of Health Research (CIHR) master's degree and FRQS postdoctoral scholarships. The Chronic Pain Epidemiology Laboratory led by AL was funded by the Fondation de l'Université du Québec en Abitibi-Témiscamingue (FUQAT), in partnership with local businesses: the Pharmacie Jean-Coutu de Rouyn-Noranda (community pharmacy) and Glencore Fonderie Horne (copper smelter). LB received research grants from AstraZeneca, TEVA and Genentech, as well as consultation fees from AstraZeneca, TEVA and Genentech for projects unrelated to this study. The remaining authors declare that the research was conducted in the absence of any commercial or financial relationships that could be construed as a potential conflict of interest.

## Publisher's Note

All claims expressed in this article are solely those of the authors and do not necessarily represent those of their affiliated organizations, or those of the publisher, the editors and the reviewers. Any product that may be evaluated in this article, or claim that may be made by its manufacturer, is not guaranteed or endorsed by the publisher.

## References

[1] Argoff CE Albrecht P Irving G Rice F. multimodal analgesia for chronic pain: rationale and future directions. Pain Med. (2009) 10 (Suppl. 2):S53–66. 10.1111/j.1526-4637.2009.00669.x 19691685

[2] Turk DC Swanson KS Tunks ER. Psychological approaches in the treatment of chronic pain patients–when pills, scalpels, and needles are not enough. Can J Psychiatry. (2008) 53:213–23. 10.1177/070674370805300402 18478824

[3] Canadian Pain Task Force. Chronic Pain in Canada: Laying a Foundation for Action. Ottawa: Health Canada (2019).

[4] Andersson HI Ejlertsson G Leden I Schersten B. Impact of chronic pain on health care seeking, self care, and medication. Results from a population-based Swedish study. J Epidemiol Community Health. (1999) 53:503–9. 10.1136/jech.53.8.503 10562870 PMC1756941

[5] Toblin RL Mack KA Perveen G Paulozzi LJ. A population-based survey of chronic pain and its treatment with prescription drugs. Pain. (2011) 152:1249–55. 10.1016/j.pain.2010.12.036 21397401

[6] Choiniere M Dion D Peng P Banner R Barton PM Boulanger A. The Canadian stop-pain project - part 1: who are the patients on the waitlists of multidisciplinary pain treatment facilities? Can J Anaesth. (2010) 57:539–48. 10.1007/s12630-010-9305-5 20393821

[7] Mazzotti E Antonini Cappellini GC Buconovo S Morese R Scoppola A Sebastiani C. Treatment-related side effects and quality of life in cancer patients. Supportive Care Cancer. (2012) 20:2553–7. 10.1007/s00520-011-1354-y 22270087

[8] Timmerman L Stronks DL Groeneweg JG Huygen FJ. Prevalence and determinants of medication non-adherence in chronic pain patients: a systematic review. Acta anaesthesiologica Scandinavica. (2016) 60:416–31. 10.1111/aas.12697 26860919

[9] Mayo-Wilson E Golozar A Cowley T Fusco N Gresham G Haythornthwaite J. Methods to identify and prioritize patient-centered outcomes for use in comparative effectiveness research. Pilot Feasibility Stud. (2018) 4:95. 10.1186/s40814-018-0284-6 30026961 PMC6047482

[10] Treweek S Zwarenstein M. Making trials matter: pragmatic and explanatory trials and the problem of applicability. Trials. (2009) 10:37. 10.1186/1745-6215-10-37 19493350 PMC2700087

[11] Strom BL Kimmel SE Hennessy S. Pharmacoepidemiology. 5th ed. Sussex: Wiley-Blackwell (2012).

[12] Gordis L. Epidemiology. 5 ed. Philadelphia: Elsevier Saunders (2014).

[13] Travers J Marsh S Caldwell B Williams M Aldington S Weatherall M. External validity of randomized controlled trials in copd. Respir Med. (2007) 101:1313–20. 10.1016/j.rmed.2006.10.011 17113277

[14] Travers J Marsh S Williams M Weatherall M Caldwell B Shirtcliffe P. External validity of randomised controlled trials in asthma: to whom do the results of the trials apply? Thorax. (2007) 62:219–23. 10.1136/thx.2006.066837 17105779 PMC2117157

[15] Liu KA Mager NAD. Women's involvement in clinical trials: historical perspective and future implications. Pharm Pract. (2016) 14:708. 10.18549/PharmPract.2016.01.708 27011778 PMC4800017

[16] Berlach DM Shir Y Ware MA. Experience with the synthetic cannabinoid nabilone in chronic noncancer pain. Pain Med. (2006) 7:25–9. 10.1111/j.1526-4637.2006.00085.x 16533193

[17] Fitzcharles MA Shir Y. Management of chronic pain in the rheumatic diseases with insights for the clinician. Therap Adv Musculosk Dis. (2011) 3:179–90. 10.1177/1759720X11408999 22870477 PMC3382677

[18] Giladi H Choiniere M Fitzcharles MA Ware MA Tan X Shir Y. Pregabalin for chronic pain: does one medication fit all? Curr Med Res Opin. (2015) 31:1403–11. 10.1185/03007995.2015.1040750 25868712

[19] Eguale T Buckeridge DL Winslade NE Benedetti A Hanley JA Tamblyn R. Drug, patient, and physician characteristics associated with off-label prescribing in primary care. Arch Intern Med. (2012) 172:781–8. 10.1001/archinternmed.2012.340 22507695

[20] Fitzcharles MA Ste-Marie PA Goldenberg DL Pereira JX Abbey S Choiniere M. 2012 Canadian guidelines for the diagnosis and management of fibromyalgia syndrome: executive summary. Pain Res Manag. (2013) 18:119–26. 10.1155/2013/918216 23748251 PMC3673928

[21] Attal N Cruccu G Baron R Haanpaa M Hansson P Jensen TS. Efns guidelines on the pharmacological treatment of neuropathic pain: 2010 revision. Eur J Neurol. (2010) 17:1113–e88. 10.1111/j.1468-1331.2010.02999.x 20402746

[22] Menditto E Gimeno Miguel A Moreno Juste A Poblador Plou B Aza Pascual-Salcedo M Orlando V. Patterns of multimorbidity and polypharmacy in young and adult population: systematic associations among chronic diseases and drugs using factor analysis. PLoS ONE. (2019) 14:e0210701. 10.1371/journal.pone.0210701 30726245 PMC6364882

[23] CIHR. How to Integrate Sex and Gender into Research. Ottawa: Institute of Gender and Health, Canadian Institutes of Health Research (2018). Available online at: http://www.cihr-irsc.gc.ca/e/50836.html (accessed June 26, 2018).

[24] Johnson JL Greaves L Repta R. Better science with sex and gender: facilitating the use of a sex and gender-based analysis in health research. Int J Equity Health. (2009) 8:1–11. 10.1186/1475-9276-8-14 19419579 PMC2689237

[25] Fillingim RB Ness TJ Glover TL Campbell CM Hastie BA Price DD. Morphine responses and experimental pain: sex differences in side effects and cardiovascular responses but not analgesia. J Pain. (2005) 6:116–24. 10.1016/j.jpain.2004.11.005 15694878

[26] Lopes GS Bielinski S Moyer AM Jacobson DJ Wang L Jiang R. Sex Differences in type and occurrence of adverse reactions to opioid analgesics: a retrospective cohort study. BMJ Open. (2021) 11:e044157. 10.1136/bmjopen-2020-044157 34193479 PMC8246359

[27] Daoust R Paquet J Lavigne G Piette E Chauny JM. Impact of age, sex and route of administration on adverse events after opioid treatment in the emergency department: a retrospective study. Pain Res Manag. (2015) 20:23–8. 10.1155/2015/316275 25664538 PMC4325886

[28] Sagy I Friger M Sagy TP Rudich Z. Gender-based differences in the management of low back pain. Harefuah. (2014) 153:380-4:434. 25189025

[29] Lacasse A Gagnon V Nguena Nguefack HL Gosselin M Pagé MG Blais L. Chronic pain patients' willingness to share personal identifiers on the web for the linkage of medico-administrative claims and patient-reported data: the chronic pain treatment cohort. Pharmacoepidemiol Drug Saf. (2021) 30:1012–26. 10.1002/pds.5255 33901339 PMC8360172

[30] Treede RD Rief W Barke A Aziz Q Bennett MI Benoliel R. Chronic pain as a symptom or a disease: the iasp classification of chronic pain for the international classification of diseases (Icd-11). Pain. (2019) 160:19–27. 10.1097/j.pain.0000000000001384 30586067

[31] Sauvé J-S. L'interdiction De Discriminer Les Personnes Trans^*^ Dans La Charte Des Droits Et Libertés De La Personne : Pour Son Amélioration Par L'ajout De L'≪ identité De Genre ≪ Et De L'≪ expression De Genre ≫ À La Liste Des Motifs De Distinction Illicites. Enfances Familles Générations. (2015) 2015:108–26. 10.7202/1034203ar

[32] Rose R. Les Femmes Et Le Marché Du Travail Au Québec : Portrait Statistique [Women and the Labour Market in Quebec: A Statistical Portrait]. Montreal: Comité consultatif Femmes en développement de la main-d'oeuvre (2016).

[33] Descarries F. Le Mouvement Des Femmes Québecois: État Des Lieux. Cités. (2005) 23:143–54. 10.3917/cite.023.0143

[34] Conseil du statut de la femme. Portrait Des Québécoises. Édition 2020 – Femmes Et Économie [Portrait of Quebec Women. 2020 Edition - Women and the Economy]. Québec: Conseil du statut de la femme, (2020).

[35] Gervais C de Montigny F Lavoie K Garneau J Dubeau D. Conceptions and experiences of paternal involvement among quebec fathers: a dual parental experience. J Fam Issues. (2021) 42:374–94. 10.1177/0192513X20910174

[36] Ledoux C Thuillier B. Du Travail Domestique Masculine Au Travail Domestique Des Hommes. (Analyse Quantitative). Terrains & travaux. (2006) 10:56–76. 10.3917/tt.010.0056

[37] CPN. Chronic Pain Network Annual Report 2016/2017. Hamilton, ON: Chronic Pain Network (2017).

[38] Turk DC Dworkin RH Allen RR Bellamy N Brandenburg N Carr DB. Core outcome domains for chronic pain clinical trials: immpact recommendations. Pain. (2003) 106:337–45. 10.1016/j.pain.2003.08.001 14659516

[39] Dworkin RH Turk DC Farrar JT Haythornthwaite JA Jensen MP Katz NP. Core outcome measures for chronic pain clinical trials: immpact recommendations. Pain. (2005) 113:9–19. 10.1016/j.pain.2004.09.012 15621359

[40] Lacasse A Roy JS Parent AJ Noushi N Odenigbo C Page G. The Canadian minimum dataset for chronic low back pain research: a cross-cultural adaptation of the national institutes of health task force research standards. CMAJ Open. (2017) 5:E237–E48. 10.9778/cmajo.20160117 28401140 PMC5378521

[41] Choiniere M Ware MA Page MG Lacasse A Lanctot H Beaudet N. Development and implementation of a registry of patients attending multidisciplinary pain treatment clinics: the Quebec pain registry. Pain Res Manag. (2017) 2017:1–16. 10.1155/2017/8123812 28280406 PMC5322414

[42] Moulin DE Clark AJ Gordon A Lynch M Morley-Forster PK Nathan H. Long-term outcome of the management of chronic neuropathic pain: a prospective observational study. J Pain. (2015) 16:852–61. 10.1016/j.jpain.2015.05.011 26080044

[43] Deyo RA Dworkin SF Amtmann D Andersson G Borenstein D Carragee E. Report of the Nih task force on research standards for chronic low back pain. J Pain. (2014) 15:569–85. 10.1016/j.jpain.2014.03.005 24787228 PMC4128347

[44] Goodman M Adams N Corneil T Kreukels B Motmans J Coleman E. Size and distribution of transgender and gender nonconforming populations: a narrative review. Endocrinol Metab Clin North Am. (2019) 48:303–21. 10.1016/j.ecl.2019.01.001 31027541

[45] Statistics Canada. Sexual Minority People Almost Three Times More Likely to Experience Violent Victimization Than Heterosexual People. Ottawa: Statistics Canada (2020).

[46] Bem SL. The measurement of psychological androgyny. J Consult Clin Psychol. (1974) 42:155–62. 10.1037/h0036215 4823550

[47] Fontayne P Sarrazin P Famose J-P. The Bem sex-role inventory: validation of a short version for French teenagers. Eur Rev Appl Psychol. (2000) 50:405–16. Available online at: https://hal.archives-ouvertes.fr/file/index/docid/387229/filename/Fontayne_etal_ERAP2000.pdf (accessed April 25, 2022).

[48] Hoffman RM Borders LD. Twenty-five years after the Bem sex-role inventory: a reassessment and new issues regarding classification variability. Measure Evaluat Counsel Dev. (2001) 34:39–55. 10.1080/07481756.2001.12069021

[49] DeCoster J Gallucci M Iselin A-MR. Best practices for using median splits, artificial categorization, and their continuous alternatives. J Exp Psychopathol. (2011) 2:197–209. 10.5127/jep.008310

[50] Blackman S. Comments on three methods of scoring androgyny as a continuous variable. Psychol Rep. (1982) 51 (3_suppl):1100–2. 10.2466/pr0.1982.51.3f.1100

[51] Bruyere O Demoulin M Brereton C Humblet F Flynn D Hill JC. Translation validation of a new back pain screening questionnaire (the start back screening tool) in French. Arch Public Health. (2012) 70:12. 10.1186/0778-7367-70-12 22958224 PMC3436683

[52] Bouhassira D Attal N Alchaar H Boureau F Brochet B Bruxelle J. Comparison of pain syndromes associated with nervous or somatic lesions and development of a new neuropathic pain diagnostic questionnaire (Dn4). Pain. (2005) 114:29–36. 10.1016/j.pain.2004.12.010 15733628

[53] Cleeland CS. The Brief Pain Inventory User Guide. Houston, TX: The University of Texas MD Anderson Cancer Center (2009).

[54] Assayag J Forget A Kettani FZ Beauchesne MF Moisan J Blais L. The impact of the type of insurance plan on adherence and persistence with antidepressants: a matched cohort study. Can J Psychiatry. (2013) 58:233–9. 10.1177/070674371305800409 23547647

[55] Heidari S Babor TF De Castro P Tort S Curno M. Sex and gender equity in research: rationale for the sager guidelines and recommended use. Res Integr Peer Rev. (2016) 1:2. 10.1186/s41073-016-0007-6 29451543 PMC5793986

[56] Canadian Institutes of Health Research. Online Training Modules: Integrating Sex & Gender in Health Research - Sex and Gender in the Analysis of Data from Human Participants. Ottawa: Canadian Institutes of Health Research (CIHR) (2017). Available online at: http://www.cihr-irsc.gc.ca/e/49347.html (accessed July 2, 2018).

[57] Mena E Bolte G Bolte G Mena E Rommel A Saß A-C. Intersectionality-based quantitative health research and sex/gender sensitivity: a scoping review. Int J Equity Health. (2019) 18:199. 10.1186/s12939-019-1098-8 31864366 PMC6925460

[58] Farewell V Long D Tom B Yiu S Su L. Two-part and related regression models for longitudinal data. Annual review of statistics and its application. (2017) 4:283–315. 10.1146/annurev-statistics-060116-054131 28890906 PMC5590716

[59] Sourial N Vedel I Le Berre M Schuster T. Testing group differences for confounder selection in nonrandomized studies: flawed practice. Can Med Assoc J. (2019) 191:E1189–E93. 10.1503/cmaj.190085 31659059 PMC6821495

[60] Katz MH. Multivariable Analysis: A Practical Guide for Clinicians and Public Health Researchers. Cambridge: Cambridge University Press (2011).

[61] Vatcheva KP Lee M McCormick JB Rahbar MH. Multicollinearity in regression analyses conducted in epidemiologic studies. Epidemiology. (2016) 6:227. 10.4172/2161-1165.1000227 27274911 PMC4888898

[62] Pieretti S Di Giannuario A Di Giovannandrea R Marzoli F Piccaro G Minosi P. Gender differences in pain and its relief. Annali dell'Istituto superiore di sanita. (2016) 52:184–9. 10.4415/ANN_16_02_09 27364392

[63] Boerner KE Chambers CT Gahagan J Keogh E Fillingim RB Mogil JS. Conceptual complexity of gender and its relevance to pain. Pain. (2018) 159:2137–41. 10.1097/j.pain.0000000000001275 29781962

[64] Fillingim RB King CD Ribeiro-Dasilva MC Rahim-Williams B Riley III JL. Sex, gender, and pain: a review of recent clinical and experimental findings. J Pain. (2009) 10:447–85. 10.1016/j.jpain.2008.12.001 19411059 PMC2677686

[65] Welch V Doull M Yoganathan M Jull J Boscoe M Coen SE. Reporting of sex and gender in randomized controlled trials in canada: a cross-sectional methods study. Res Integrity Peer Rev. (2017) 2:15. 10.1186/s41073-017-0039-6 29451565 PMC5803639

[66] Moride Y Abenhaim L. Evidence of the depletion of susceptibles effect in non-experimental pharmacoepidemiologic research. J Clin Epidemiol. (1994) 47:731–7. 10.1016/0895-4356(94)90170-8 7722586

[67] Bijur PE Esses D Birnbaum A Chang AK Schechter C Gallagher EJ. Response to morphine in male and female patients: analgesia and adverse events. Clin J Pain. (2008) 24:192–8. 10.1097/AJP.0b013e31815d3619 18287823

[68] LeGates TA Kvarta MD Thompson SM. Sex differences in antidepressant efficacy. Neuropsychopharmacology. (2019) 44:140–54. 10.1038/s41386-018-0156-z 30082889 PMC6235879

[69] Mauvais-Jarvis F Merz NB Barnes PJ Brinton RD Carrero J-J DeMeo DL. Sex and gender: modifiers of health, disease, and medicine. Lancet. (2020) 396:565–82. 10.1016/S0140-6736(20)31561-0 32828189 PMC7440877

[70] Fernández-Liz E Modamio P Catalán A Lastra CF Rodríguez T Mariño EL. Identifying how age and gender influence prescription drug use in a primary health care environment in Catalonia, Spain. Br J Clin Pharmacol. (2008) 65:407–17. 10.1111/j.1365-2125.2007.03029.x 17922886 PMC2291251

[71] Isacson D Bingefors K. Epidemiology of analgesic use: a gender perspective. Eur J Anaesthesiol. (2001) 19:5–15. 10.1097/00003643-200219261-00003 12512211

[72] Anderson GD. Gender differences in pharmacological response. Int Rev Neurobiol. (2008) 83:1–10. 10.1016/S0074-7742(08)00001-9 18929073

[73] Montejo-gonzàlez AL Llorca G Izquierdo JA Ledesma A Bousono M Calcedo A. Fluoxetine, paroxetine, sertraline, and fluvoxamine in a prospective, multicenter, and descriptive clinical study of 344 patients. J Sex Marital Ther. (1997) 23:176–94. 10.1080/00926239708403923 9292833

[74] Susan G Kornstein MD Alan F Schatzberg MD Michael E Thase MD. Gender differences in treatment response to sertraline versus imipramine in chronic depression. Am J Psychiatry. (2000) 157:1445–52. 10.1176/appi.ajp.157.9.1445 10964861

[75] Franconi F Campesi I Occhioni S Antonini P Murphy MF. Sex and gender in adverse drug events, addiction, and placebo. Handb Exp Pharmacol. (2013) 214:107–26. 10.1007/978-3-642-30726-3_6 23027448

[76] Lengua LJ Stormshak EA. Gender, gender roles, and personality: gender differences in the prediction of coping and psychological symptoms. Sex Roles. (2000) 43:787–820. 10.1023/A:101109660486120711893

[77] Lopez-Martinez AE Esteve-Zarazaga R Ramirez-Maestre C. Perceived social support and coping responses are independent variables explaining pain adjustment among chronic pain patients. J Pain. (2008) 9:373–9. 10.1016/j.jpain.2007.12.002 18203665

[78] Nascimento MG Kosminsky M Chi M. Gender role in pain perception and expression: an integrative review. BrJP. (2020) 3:58–62. 10.5935/2595-0118.20200013

[79] Richardson J Holdcroft A. Gender differences and pain medication. Womens Health. (2009) 5:79–88. 10.2217/17455057.5.1.79 19102644

[80] Gatchel RJ Peng YB Peters ML Fuchs PN Turk DC. The biopsychosocial approach to chronic pain: scientific advances and future directions. Psychol Bull. (2007) 133:581–624. 10.1037/0033-2909.133.4.581 17592957

[81] CEFRIO. L'usage Des Médias Sociaux Au Québec. Enquête NETendances. (2018) 9:1–18.

[82] Marshall K. Utilisation De L'ordinateur Au Travail. L'emploi et le revenu en perspective - L'édition en ligne. (2001) 2:1–8.

[83] Loikas D Wettermark B von Euler M Bergman U Schenck-Gustafsson K. Differences in drug utilisation between men and women: a cross-sectional analysis of all dispensed drugs in Sweden. BMJ Open. (2013) 3:e002378. 10.1136/bmjopen-2012-002378 23645921 PMC3646185

[84] Sedney MA. Comments on median split procedures for scoring androgyny measures. Sex Roles. (1981) 7:217–22. 10.1007/BF00287807

