# Supplementary material for: Gender Differences in Medication Adverse Effects Experienced by People Living With Chronic Pain
Source: Front Pain Res (Lausanne). 2022 May 10;3:830153. doi: 10.3389/fpain.2022.830153 (PMC9128021; doi:10.3389/fpain.2022.830153)
Supplement: Supplementary file 1 [file Data_Sheet_1.docx]

Supplementary Material

**Supplemental Digital Content 1**

A multivariable two-part regression model (Farewell et al., 2017) was used to assess the association between gender identity and gender roles (independent variables), and the number of adverse effects reported as severe by participants (dependent variable). Such count data are commonly modelled using Poisson or negative binomial regression (Katz, 2011; Rose et al., 2006). However, when data are zero-inflated (many participants with values = 0), those models should be replaced by zero-inflated approaches (Zero-Inflated Poisson [ZIP], Zero-Inflated Negative Binomial) (Hu et al., 2011; Yang, 2014), which are two-part models (Ridout et al., 2001).Those models provide one method to explain the excess zeros by modelling the data as a mixture of two separate distributions: one is a constant distribution that generates only zero counts (called structural zero), and the other is generally a Poisson or negative binomial distribution that can generate both zero (called sampling zero) and nonzero counts (Hu et al., 2011; Ridout et al., 1998; Ridout et al., 2001). Hence, the first part of zero-inflated models can be modelled using logistic regression, and the second part using Poisson or negative binomial regression (Yusuf et al., 2017), depending on overdispersion (Yang, 2014; Yusuf et al., 2017).

As our outcome of interest (number of adverse effects reported as severe) was zero-inflated and showed almost no overdispersion (dispersion parameter = 1.22, which is less than the overdispersion cut-off of 2 for Poisson distribution (Commenges & Jacqmin-Gadda, 2015)), a ZIP model was used (SAS® proc genmod). In the first part, logistic regression was applied to assess the association between gender identity, gender, covariates, and the probability of having structural zero adverse effects reported as severe. In other words, this part answered the clinical question, “Who has the greater likelihood of experiencing no adverse effects reported as severe whatsoever?” As for the second part, Poisson regression was used with the same covariates to find the relevant predictors of the number of adverse effects reported as severe among those having non structural zero adverse effects reported as severe (“Who has the greater likelihood of experiencing a greater number of severe adverse effects?”). Results of the first part of the model (logistic regression) were computed as adjusted odds ratios (OR) along with their respective 95% CI and *p*-values; results from the second part of the model (Poisson regression) were computed as adjusted beta coefficients (ß) along with their respective 95% CI and *p*-values.

**Supplemental Digital Content 2**

**Multivariable model exploring associations between gender identity, gender roles and number of severe adverse effects.**

|  | Odds Ratio for the Probability of having structural zero severe adverse effect | | | Coefficient Estimates for the number of severe adverse effects among participants with nonstructural zero severe adverse effect | | |
| --- | --- | --- | --- | --- | --- | --- |
|  | Adjusted OR | 95% CI | p-value | Adjusted ß | 95% CI | p-value |
| Sex (men vs. women) | 1.04 | 0.48-2.24 | 0.9276 | **-0.32** | **-0.52- -0.11** | **0.0024** |
| Gender (vs. Undifferentiated) |  |  |  |  |  |  |
| Feminine | 0.60 | 0.30-1.22 | 0.1570 | 0.06 | -0.11-0.23 | 0.4821 |
| Masculine | 1.19 | 0.59-2.41 | 0.6271 | 0.03 | -0.19-0.25 | 0.7938 |
| Androgynous | 1.08 | 0.58-2.00 | 0.8084 | **0.26** | **0.09-0.44** | **0.0030** |
| Back pain (Yes vs. No) | 1.03 | 0.55-1.95 | 0.9246 | -0.15 | -0.35-0.06 | 0.1633 |
| Neck pain (Yes vs. No) | 1.94 | 0.99-3.79 | 0.0527 | **0.20** | **0.00-0.39** | **0.0456** |
| Shoulder pain (Yes vs. No) | 0.66 | 0.35-1.25 | 0.2010 | 0.03 | -0.17-0.22 | 0.7831 |
| Leg pain (Yes vs. No ) | 0.85 | 0.47-1.53 | 0.5883 | 0.05 | -0.12-0.23 | 0.5566 |
| Hip pain (Yes vs. No ) | 0.98 | 0.56-1.71 | 0.9499 | -0.14 | -0.31-0.04 | 0.1305 |
| Generalized pain (Yes vs. No ) | 1.06 | 0.61-1.85 | 0.8388 | **0.17** | **0.02-0.32** | **0.0270** |
| Multisite pain (Yes vs. No ) | 0.72 | 0.30-1.73 | 0.4619 | -0.22 | -0.51-0.08 | 0.1459 |
| Onset of pain following an accident (Yes vs. No ) | 0.87 | 0.50-1.50 | 0.6103 | -0.11 | -0.25-0.04 | 0.1470 |
| Onset of pain following stressful event (Yes vs. No ) | 1.42 | 0.81-2.49 | 0.2262 | 0.10 | -0.05-0.25 | 0.1858 |
| Onset of pain following diseases (Yes vs. No ) | 0.96 | 0.58-1.58 | 0.8781 | **-0.18** | **-0.32- -0.05** | **0.0069** |
| Pain frequency (occasionally/continuously) | 0.99 | 0.65-1.51 | 0.9607 | 0.08 | -0.08-0.25 | 0.3306 |
| Pain duration (< 5 years) |  |  |  |  |  |  |
| 5- 9 years | 0.95 | 0.48-1.88 | 0.8845 | 0.04 | -0.14-0.22 | 0.6646 |
| ≥10 years | 1.36 | 0.76-2.46 | 0.3012 | -0.01 | -0.18-0.16 | 0.9074 |
| Tendency to pain catastrophizing (Yes vs. No ) | **0.75** | **0.56-0.99** | **0.0430** | -0.00 | -0.09-0.09 | 0.9523 |
| Evidence of neuropathic pain (Yes vs. No ) | 0.67 | 0.41-1.10 | 0.1159 | **0.27** | **0.13-0.42** | **0.0002** |
| Brief Pain Inventory (BPI) score (continuous) | 0.95 | 0.80-1.12 | 0.5180 | **0.13** | **0.08-0.18** | **<0.0001** |
| Pain intensity on average in the past 7 days (continuous) | 1.11 | 0.96-1.29 | 0.1743 | **0.08** | **0.03-0.12** | **0.0006** |
| Pharmacological pain treatments (vs. Over-the-counter medications only) |  |  |  |  |  |  |
| Prescribed medications only | **0.28** | **0.12-0.63** | **0.0021** | **0.53** | **0.08-0.98** | **0.0205** |
| Both | **0.23** | **0.10- 0.50** | **0.0002** | **0.45** | **0.01-0.90** | **0.0453** |
| Non-pharmacological treatments (Yes vs. No) | 0.83 | 0.61-1.11 | 0.1999 | -0.03 | -0.11-0.05 | 0.4008 |
| Access to a trusted healthcare professional for pain management (Yes vs. No) | 0.76 | 0.58-1.00 | 0.0536 | -0.05 | -0.13-0.03 | 0.2582 |
| Percentage of relief provided by pain treatments (continuous) | 0.99 | 0.98-1.01 | 0.2706 | -0.00 | -0.00-0.00 | 0.5879 |
| Country of birth (Canada vs. Others) | 0.55 | 0.19-1.56 | 0.2601 | -0.04 | -0.38-0.31 | 0.8255 |
| Employment (Working vs. Not working) | 0.80 | 0.43-1.47 | 0.4670 | 0.03 | -0.16-0.22 | 0.7616 |
| Temporary or permanent disability (Yes vs. No) | 1.23 | 0.67-2.23 | 0.5031 | **0.24** | **0.08-0.40** | **0.0029** |
| Education level (vs. Secondary education or less) |  |  |  |  |  |  |
| Post-secondary education | 1.59 | 0.79-3.18 | 0.1950 | **0.16** | **0.01-0.32** | **0.0423** |
| University education | **2.85** | **1.38-5.89** | **0.0048** | 0.13 | -0.05-0.30 | 0.1546 |
| Living in a remote region (Yes vs. No) | 1.06 | 0.63-1.78 | 0.8320 | 0.15 | -0.01-0.30 | 0.0583 |
| Age (continuous) | 0.99 | 0.97-1.01 | 0.3771 | **0.01** | **0.00-0.01** | **0.0444** |
| Physical Functioning (PF) score (continuous) | 1.02 | 0.99-1.05 | 0.2152 | **0.01** | **0.00-0.02** | **0.0478** |
| General Health (GH) score (continuous) | 1.02 | 1.00-1.04 | 0.1146 | -0.00 | -0.01-0.00 | 0.3031 |
| Polypharmacy/Number of pain medication (continuous) | **0.91** | **0.85- 0.97** | **0.0049** | **0.02** | **0.00-0.03** | **0.0178** |
| Patient Health Questionnaire-4 score (vs. None) |  |  |  |  |  |  |
| Mild | 0.95 | 0.47-1.91 | 0.8834 | **0.43** | **0.18-0.68** | **0.0008** |
| Moderate | 0.75 | 0.34-1.66 | 0.4734 | **0.33** | **0.06-0.59** | **0.0161** |
| Severe | 0.53 | 0.21-1.38 | 0.1965 | **0.41** | **0.14-0.69** | **0.0028** |
| Need to reduce alcohol or drugs consumption (vs. Never) |  |  |  |  |  |  |
| Rarely | 1.32 | 0.68-2.54 | 0.4076 | -0.01 | -0.21-0.20 | 0.9637 |
| Sometimes | 1.38 | 0.61-3.13 | 0.4457 | 0.14 | -0.11-0.39 | 0.2732 |
| Often | 1.19 | 0.53-2.66 | 0.6768 | 0.18 | -0.06-0.41 | 0.1417 |
| Cannabis for pain management (Yes vs. No) | 0.55 | 0.28-1.10 | 0.0934 | -0.07 | -0.24-0.09 | 0.3798 |
| Cannabis for the management of other health problems (Yes vs. No) | 1.40 | 0.59-3.33 | 0.4422 | **0.23** | **0.01-0.45** | **0.0440** |
| Cannabis for recreational purposes (Yes vs. No) | 1.46 | 0.66-3.23 | 0.3475 | -0.10 | -0.32-0.13 | 0.4002 |
| Smoking (vs. Never smoked) |  |  |  |  |  |  |
| Current smoker | 1.16 | 0.55-2.44 | 0.6929 | 0.10 | -0.07-0.27 | 0.2629 |
| Used to smoke, but have now quit | 1.07 | 0.64-1.77 | 0.8066 | -0.01 | -0.15-0.14 | 0.9381 |

*Table footnotes:*

P-values <.05 are reported in bold

**Supplemental Digital Content 3**

**Multivariable model exploring associations between gender identity, gender roles and number of severe adverse effects using imputed data.**

| Coefficients Estimates for the number of severe adverse effects among participants with non structural zero severe adverse effect | | | | |
| --- | --- | --- | --- | --- |
| **Model without interaction terms** | | | | |
|  | Adjusted ß^*^ | 95% CI | p-value |  |
| **Gender identity** (men vs. women) | **-0.22** | **-0.40- -0.05** | **0.0116** |  |
| **Gender** (vs. Undifferentiated) |  |  |  |  |
| Feminine (describe themselves as tender and sensitive to others) | 0.07 | -0.07-0.20 | 0.3483 |  |
| Masculine (describe themselves as athletic, having leadership, and being self-confident) | -0.03 | -0.22-0.17 | 0.7977 |  |
| Androgynous (scored high on all these traits) | **0.16** | **0.01-0.31** | **0.0363** |  |
| **Model with interaction terms** | | | | |
| **Gender identity** (men vs. women) | **-0.37** | **-0.68- -0.05** | **0.0221** |  |
| **Gender** (vs. Undifferentiated) |  |  |  |  |
| Feminine | -0.01 | -0.13-0.15 | 0.8818 |  |
| Masculine | 0.04 | -0.16-0.24 | 0.6799 |  |
| Androgynous | **0.15** | **0.00-0.31** | **0.0497** |  |
| Interaction terms |  |  |  |  |
| *Gender identity * Feminine* | **0.55** | **0.12- -0.98** | **0.0121** |  |
| *Gender identity * Masculine* | -0.27 | -0.83-0.29 | 0.3413 |  |
| *Gender identity * Androgynous* | 0.06 | -0.34-0.45 | 0.7722 |  |

*Table footnotes:*

P-values <.05 are reported in bold

* Adjusted for circumstances surrounding onset of pain, pain location, frequency, duration, tendency to pain catastrophizing, evidence of neuropathic pain, Brief Pain Inventory (BPI) score, pharmacological pain treatment use, non-pharmacological treatment use, access to a trusted healthcare professional for pain management, percentage of relief provided by pain treatment, country of birth, employment, disability, education level, living in a remote region, age, physical functioning score, general health score, number of drugs used, Patient Health Questionnaire-4 score, alcohol or drugs perceived problem, cannabis use, and smoking; 976 participants with no missing data were included in the final model.

**References**

Commenges, D., & Jacqmin-Gadda, H. (2015). *Modèles biostatistiques pour l'épidémiologie*. De Boeck Superieur.

Farewell, V., Long, D., Tom, B., Yiu, S., & Su, L. (2017). Two-part and related regression models for longitudinal data. *Annual review of statistics and its application, 4*, 283-315.

Hu, M.-C., Pavlicova, M., & Nunes, E. V. (2011). Zero-inflated and hurdle models of count data with extra zeros: examples from an HIV-risk reduction intervention trial. *The American journal of drug and alcohol abuse, 37*(5), 367-375.

Katz, M. H. (2011). *Multivariable analysis: a practical guide for clinicians and public health researchers*. Cambridge university press.

Ridout, M., Demétrio, C., & Hinle, J. (1998, 01/01). Models for count data with many zeros. International Biomeric Conference. *Cape Town, 13*, 1-13.

Ridout, M., Hinde, J., & Demétrio, C. G. (2001). A score test for testing a zero‐inflated Poisson regression model against zero‐inflated negative binomial alternatives. *Biometrics, 57*(1), 219-223.

Rose, C. E., Martin, S. W., Wannemuehler, K. A., & Plikaytis, B. D. (2006). On the use of zero-inflated and hurdle models for modeling vaccine adverse event count data. *Journal of biopharmaceutical statistics, 16*(4), 463-481.

Yang, S. (2014). A comparison of different methods of zero-inflated data analysis and its application in health surveys.

Yusuf, O., Bello, T., & Gureje, O. (2017). Zero inflated poisson and zero inflated negative binomial models with application to number of falls in the elderly. *Biostatistics and Biometrics Open Access Journal, 1*(4), 69-75.
